# Text Message and Internet Support for Coronary Heart Disease Self-Management: Results From the Text4Heart Randomized Controlled Trial

**DOI:** 10.2196/jmir.4944

**Published:** 2015-10-21

**Authors:** Leila Pfaeffli Dale, Robyn Whittaker, Yannan Jiang, Ralph Stewart, Anna Rolleston, Ralph Maddison

**Affiliations:** ^1^ National Institute for Health Innovation University of Auckland Auckland New Zealand; ^2^ Department of Cardiology Auckland City Hospital Auckland New Zealand; ^3^ Department of Medicine University of Auckland Auckland New Zealand

**Keywords:** text messaging, mHealth, cellular phone, cardiovascular diseases, intervention, lifestyle change, behavior

## Abstract

**Background:**

Mobile technology has the potential to deliver behavior change interventions (mHealth) to reduce coronary heart disease (CHD) at modest cost. Previous studies have focused on single behaviors; however, cardiac rehabilitation (CR), a component of CHD self-management, needs to address multiple risk factors.

**Objective:**

The aim was to investigate the effectiveness of a mHealth-delivered comprehensive CR program (Text4Heart) to improve adherence to recommended lifestyle behaviors (smoking cessation, physical activity, healthy diet, and nonharmful alcohol use) in addition to usual care (traditional CR).

**Methods:**

A 2-arm, parallel, randomized controlled trial was conducted in New Zealand adults diagnosed with CHD. Participants were recruited in-hospital and were encouraged to attend center-based CR (usual care control). In addition, the intervention group received a personalized 24-week mHealth program, framed in social cognitive theory, sent by fully automated daily short message service (SMS) text messages and a supporting website. The primary outcome was adherence to healthy lifestyle behaviors measured using a self-reported composite health behavior score (≥3) at 3 and 6 months. Secondary outcomes included clinical outcomes, medication adherence score, self-efficacy, illness perceptions, and anxiety and/or depression at 6 months. Baseline and 6-month follow-up assessments (unblinded) were conducted in person.

**Results:**

Eligible patients (N=123) recruited from 2 large metropolitan hospitals were randomized to the intervention (n=61) or the control (n=62) group. Participants were predominantly male (100/123, 81.3%), New Zealand European (73/123, 59.3%), with a mean age of 59.5 (SD 11.1) years. A significant treatment effect in favor of the intervention was observed for the primary outcome at 3 months (AOR 2.55, 95% CI 1.12-5.84; *P*=.03), but not at 6 months (AOR 1.93, 95% CI 0.83-4.53; *P*=.13). The intervention group reported significantly greater medication adherence score (mean difference: 0.58, 95% CI 0.19-0.97; *P*=.004). The majority of intervention participants reported reading all their text messages (52/61, 85%). The number of visits to the website per person ranged from zero to 100 (median 3) over the 6-month intervention period.

**Conclusions:**

A mHealth CR intervention plus usual care showed a positive effect on adherence to multiple lifestyle behavior changes at 3 months in New Zealand adults with CHD compared to usual care alone. The effect was not sustained to the end of the 6-month intervention. A larger study is needed to determine the size of the effect in the longer term and whether the change in behavior reduces adverse cardiovascular events.

**Trial Registration:**

ACTRN 12613000901707; https://www.anzctr.org.au/Trial/Registration/TrialReview.aspx?id=364758&isReview=true (Archived by WebCite at http://www.webcitation.org/6c4qhcHKt)

## Introduction

Coronary heart disease (CHD) remains a leading cause of death [1] and an economic burden worldwide [2]. Approximately 80% of CHD is caused by modifiable risk factors, including physical inactivity, smoking, unhealthy diet, and harmful alcohol consumption [1]. Implementing lifestyle changes and adhering to prescribed medication regimens can reduce the risk of future cardiac events and aid recovery [1]. Cardiac rehabilitation (CR) is an essential part of the contemporary management of CHD [3,4] and typically involves a program of medication and risk factor education, supervised exercise training, and psychological support.

Recent meta-analyses of randomized controlled trials (RCTs) have reported that CR is associated with improvements in mortality and morbidity [5-7], favorable cholesterol profiles [6,7], changes in smoking prevalence and blood pressure [6], and positive effects on quality of life [5,7]. Despite the benefits of CR, participation rates are less than 50% in most high- and middle-income countries [8], including New Zealand [9]. In the United States, an audit of 267,427 Medicare beneficiaries found that only 18.7% of eligible patients attended one or more outpatient CR sessions [10] and only 3 of 28 European countries estimated that CR participation was greater than 50% [11].

Most CR programs are delivered face-to-face in group sessions at hospitals or community centers. Low attendance rates indicate that the center-based approach does not suit all patients [12]. Home-based CR programs have been shown to be equally effective in clinical and health-related quality of life outcomes; however, few CR programs offer a home-based alternative [12]. Telehealth CR interventions have also shown effective reductions in CHD risk factors [13]. Greater choice of delivery model could improve CR attendance. Another option to explore is mobile CR (mHealth) because mobile technology continues to be integrated into daily life and usage rates are near 100% globally [14].

Increasingly, mHealth, the use of mobile technology (eg, short message service [SMS] text messaging, video messaging, instant messaging, the Internet, apps, and voice calling) to deliver health care, is being utilized in disease prevention and management [15,16]. Text messaging, the most researched form of mHealth, can facilitate health behavior change because it allows instant and individualized health communication and reinforcement through periodic prompts and reminders [15-18]. Recently, SMS interventions have successfully improved physical activity levels [19] and medication adherence among the CHD population [20,21]; however; CR involves supporting people to make multiple lifestyle changes because many patients have more than one behavioral risk factor. There is potential to improve individuals’ overall health and reduce health care costs by targeting multiple health behaviors [22].

The aim of this study was to investigate the effectiveness of an mHealth-delivered comprehensive CR program (Text4Heart) to improve adherence to recommended lifestyle behaviors, in addition to usual care, in adults with CHD. We hypothesized that participants receiving the mHealth program would have greater adherence to lifestyle behaviors after the intervention compared to usual care alone. Secondary objectives included exploring the effects of the intervention on cardiovascular disease (CVD) risk, illness perceptions, medication adherence, self-efficacy, and anxiety and/or depression.

## Methods

### Design

We conducted a 6-month, 2-arm, parallel RCT in 123 adults diagnosed with CHD. The study received ethical approval (New Zealand Health and Disability Ethics Committee 13/NTA/06) and the protocol was registered and published before the conclusion of recruitment (ACTRN 12613000901707) [23]. The trial was developed and reported according to the CONSORT-EHEALTH statement (Multimedia Appendix 1).

### Participants

We recruited participants from 2 large metropolitan hospitals in Auckland, New Zealand. A trained researcher screened and approached eligible patients about the study before discharge from hospital after their cardiac event. Included participants were English-speaking adults with a documented diagnosis of CHD (myocardial infarction, angina, or revascularization). Although participants were not required to have computer or Internet literacy, access to the Internet (eg, at home, work, or library) was a requirement. Participants need not own a mobile phone with text messaging capability because phones were supplied for the duration of the study if necessary. Those with untreated ventricular tachycardia, severe heart failure, life-threatening coexisting disease with life expectancy less than 1 year, and/or significant exercise limitations for reasons other than CHD were excluded.

### Procedures

Eligible participants provided informed consent (see Multimedia Appendix 2) and completed a face-to-face baseline assessment in hospital, a clinic, or home setting within 4 weeks of hospital discharge. All participants received usual care, which included inpatient rehabilitation and encouragement to attend center-based CR. Traditional CR offered at the hospital recruiting sites in this study consisted of one 1-hour outpatient education program per week for 6 weeks at a hospital or community center covering a range of topics, including cardiovascular risk factors, lifestyle change, and psychosocial support. Patients also were encouraged to attend a 16-session supervised exercise program at the participating hospital or outpatient center. Participants could take part in usual care CR from point of discharge to 6 months after their heart event. In addition to usual care, the intervention group received a 24-week mHealth program sent by automated daily text messages and access to a supporting website commencing within a week of the baseline assessment. All participants were telephoned at 3-months postrandomization to collect primary outcome data. No telephone coaching was done during this follow-up call. At 6-months postrandomization, participants were seen at a clinic or in a home setting for final follow-up assessment.

### Intervention

We created and refined the Text4Heart intervention through formative and pretesting studies following the mHealth Development and Evaluation Framework [24]. Full details of the intervention, including example text messages and screenshots of the website, can be found in the published protocol [23]. In short, a theoretically framed comprehensive program of evidence-based CR guidelines [4,25,26] was delivered by text message and a supporting website over 24 weeks. The aim was to mirror current CR programs in educating patients about their cardiovascular risk factors and supporting them to make relevant lifestyle changes. Recommended lifestyle changes included stopping smoking, limiting alcohol consumption to less than 14 units of alcohol per week, eating 5 servings of fruit and vegetables per day while decreasing salt and saturated fat content, and starting and/or maintaining regular physical activity (150 minutes of moderate-to-vigorous intensity physical activity per week).

To encourage lifestyle change, the intervention was based on social cognitive theory and the key mediator of self-efficacy. Perceived self-efficacy is the extent to which people believe they can exercise control over their health behaviors [27]. With higher levels of self-efficacy, individuals can self-regulate their behavior by setting goals, creating incentives, and enlisting social support from others to maintain their motivation [28]. Self-efficacy has been shown to decline among CR nonattenders [29], which is noteworthy because higher levels of self-efficacy are linked to better clinical outcomes such as lower blood pressure and reduced hospitalizations [30].

Although targeting self-efficacy can help change lifestyle behaviors, people with CHD may need additional support to cope cognitively and emotionally with their heart event. People diagnosed with CHD can experience many negative emotions, including anxiety and depression, which can negatively impact their recovery process [31]. One way to improve coping is to modify illness perceptions and emotional representations of the disease. The Common Sense Model was also used to frame the intervention because it specifically outlines coping strategies for modifying illness perceptions and the negative emotions that arise with a health threat [32].

Participants received 7 messages per week (1 per day) and had access to a supporting website. Intervention participants also received a pedometer to self-monitor their physical activity. Messages were tailored to participants’ name and preferred time of day to receive messages. From weeks 13 to 24, the frequency of messages decreased to 5 per week. Bidirectional messaging was used because participants were prompted to text in their weekly pedometer step counts and to ask questions or for feedback on other behaviors. Reponses to step counts were automated and based on the number of steps achieved, whereas individual questions were responded to personally by the research team within 48 hours. Participants were reimbursed for any costs associated with text messaging. The supporting website was accessed using a secure log-in system and included additional information, biweekly tips from the research team via a participant blog, graphs displaying their pedometer step counts, and short video messages from role models and medical professionals [33]. No changes were made to the intervention content or delivery during the study period. All text messages were sent from a centralized server.

### Outcome Measures

The primary outcome was patient adherence to recommended health guidelines measured as a binary variable using a self-reported composite health behavior score based on the European Prospective Investigation into Cancer (EPIC)-Norfolk Prospective Population Study [34] at 6 months. We amended the study protocol shortly after recruitment began to include an additional end point: the same composite measure was used to collect the primary outcome at 3-months postrandomization during planned telephone calls because we decided it would be interesting to measure behavior change at the halfway point of the study in addition to 6 months. Participants received a score from 0 to 4 (out of 4) based on the number of health guidelines they met. Based on their score, participants were classified as adherent if they scored 3 or more out of 4 and nonadherent if they scored 2 or less. The health behaviors, scores, and outcome measures were smoking habit (1=not currently smoking; 0=had ≥1 cigarettes in past 7 days) as measured by a smoking history questionnaire [35], fruit and vegetable intake (1 indicates ≥5 servings daily; 0 indicates ≤4 servings daily) from the New Zealand Health Survey [36], alcohol intake (1 indicates ≤13 units per week; 0 indicates ≥14 units per week) as measured by the Alcohol Use Disorders Identification Test Consumption (AUDIT C) [37], and physical activity (1 indicates ≥14 units of moderate-to-vigorous activity/week; 0 indicates ≤13 units of moderate-to-vigorous activity/week) as measured by the Godin Leisure Time Physical Activity Questionnaire [38].

Secondary outcomes were evaluated at 6 months using self-completed questionnaires and clinical assessments. Clinical outcomes included individual biomedical risk factors (systolic and diastolic blood pressure, lipid profile, weight, body mass index, waist-to-hip ratio) and subsequent CHD risk probability using models proposed by D’Agostino developed from the Framingham Heart Study [39]. Medication adherence was measured using the Morisky 8-item Medication Adherence Questionnaire [40]. Psychological measures included the Self-efficacy for Managing Chronic Disease 6-item scale [41], the Brief Illness Perception Questionnaire [42], and the Hospital Anxiety and Depression Scale [43]. Serious adverse event data were collected at the 6-month assessment. Fidelity to the Text4Heart intervention was assessed using an author-derived questionnaire and calculating website and response text message usage statistics (intervention group only).

### Randomization and Blinding

Following informed consent and the baseline assessment, participants were randomized to either the intervention or the control group in a one-to-one ratio and stratified according to smoking status (smoker vs nonsmoker) to balance baseline health behavior scores. The randomization sequence was computer generated by a statistician independent to the project using a block size of 6. Allocation was concealed in sequentially numbered, opaque, sealed envelopes. Participant enrollment and assignment to the intervention were completed by a trained research assistant after baseline data collection. Because of the nature of the intervention, participants and outcome assessors were not blinded to their treatment allocation. Investigators, project statisticians, and usual care CR program leaders were blinded to group allocation.

### Analysis

#### Sample Size

We estimated that a sample size of 120 (60 per group) would provide at least 80% power at the 5% level of significance (2-sided) to detect an absolute difference of 25% between the 2 groups, in the proportions of participants adherent to recommended healthy behavior guidelines. A previous study using a similar health behavior score found that approximately 70% of adults without known cardiovascular disease adhered to 3 to 4 out of 4 health behaviors [34]. We estimated that 30% of our study population with established CHD would be adherent at baseline and hypothesized that the Text4Heart intervention would change the proportion of participants’ adherent to recommended healthy behavior guidelines by at least 25% compared to the control group at 6 months postrandomization.

#### Statistical Methods

We analyzed treatment evaluations by intention to treat, using the observed data collected from all randomized participants. Missing data were not imputed if the proportion of missing in the primary outcome was less than 10%. We did all statistical analyses using SAS version 9.3 (SAS Institute, Cary, NC, USA). All statistical tests were 2-sided at a 5% significance level. A formal statistical analysis plan was approved by the trial steering committee before data lock.

We used logistic regression to measure the main treatment effect on the proportion of participants adherent to lifestyle change (≥3 of 4 behaviors) at the end of the 6-month intervention period, adjusting for baseline adherence level and stratification factor (smoking status) and on the same outcome at 3 months. We used analysis of covariance (ANCOVA) regression to evaluate the treatment effect on continuous secondary outcomes, adjusting for baseline outcome value (if measured) and smoking status. We completed frequency and descriptive statistics on the intervention feedback survey and website and response text message usage statistics using Microsoft Excel 2010. All analyses on secondary outcomes were exploratory. We did not consider any sensitivity or subgroup analyses.

## Results

**Figure 1:**
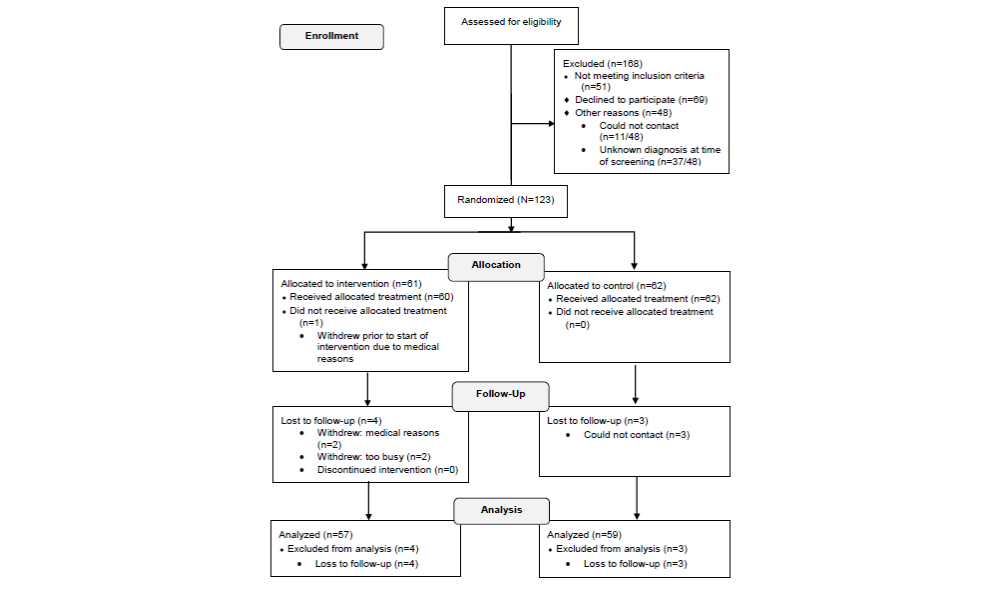
Trial registration flowchart.

Figure 1 presents the flow diagram of the progress through the phases of the trial. A total of 291 patients were screened and recruited over 10 months between 2013 and 2014. Of these, 123 eligible participants were randomized to the intervention (n=61) or the control (n=62) group.

Participants were predominantly male (100/123, 81.3%), New Zealand European (73/123, 59.3%), with a mean age of 59.5 (SD 11.1) years (see Table 1 for demographics). One quarter of participants had a household income of less than the average yearly income of NZ $50,000 (31/123, 25.2%) [44]. By the 6-month assessment, approximately half had attended at least one session of usual care CR (intervention: 30/61, 49%; control: 34/62, 55%). All participants in the intervention group used their own mobile phone.

**Table 1:** Participant baseline demographic and clinical characteristics (N=123).

Characteristic		Intervention (n=61)	Control (n=62)
Age (years), mean (SD)		59.0 (10.5)	59.9 (11.8)
**Gender, n (%)**			
	Male	48 (79)	52 (84)
	Female	13 (21)	10 (16)
**Ethnicity** ^a^ **, n (%)**			
	New Zealand or other European	46 (75)	45 (73)
	Māori (indigenous)	6 (10)	2 (3)
	Pacific Island	5 (8)	2 (3)
	Indian	6 (10)	8 (13)
	Other	2 (3)	5 (8)
**Income (NZ$)** ^b^ **, n (%)**			
	<50,000/year	14 (23)	17 (27)
	>50,000/year	39 (64)	40 (65)
	Don’t know/refuse to answer	8 (13)	5 (8)
**Cardiac diagnosis, n (%)**			
	Myocardial infarction	46 (75)	52 (84)
	Unstable angina	4 (7)	5 (8)
	Angina	11 (18)	5 (8)
**Cardiac procedure, n (%)**			
	Percutaneous coronary intervention	43 (70)	47 (76)
	Coronary artery bypass grafting	14 (23)	10 (16)
	Medical management	4 (7)	5 (8)
Diabetes		14 (23)	7 (11)

^a^Could identify with more than 1 ethnicity.

^b^Income split into categories based on earning less or greater than the average yearly income of NZ $50,000.

For the primary outcome, the intervention group increased adherence to recommended lifestyle behavior changes from 33% (20/61) at baseline to 59% (36/61) at 3 months and then plateaued with 53% (32/61) still adherent at 6 months. The control group had a smaller increase in adherence from 27% (17/62) at baseline to 37% (23/62) at 3 months and 39% (24/62) at 6 months. A significant treatment effect in favor of the intervention was observed at 3 months (AOR 2.55, 95% CI 1.12-5.84; *P*=.03), but not at 6 months (AOR 1.93, 95% CI 0.83-4.53; *P*=.13). The percentage adherent to individual behaviors can be seen in Table 2.

For the secondary outcomes (Table 3), the intervention group reported a significantly greater medication adherence score (mean difference: 0.58, 95% CI 0.19-0.97; *P*=.004). The intervention group also had lower low-density lipoprotein (LDL) cholesterol than the control group (mean difference: –0.25, 95% CI –0.49 to 0.01; *P*=.05) at 6 months, although this did not meet statistical significance. A negative effect was seen for total hospital anxiety with the intervention group reporting significantly greater anxiety than the control group at 6 months (mean difference: 1.18, 95% CI 0.28-2.08; *P*=.01). No differences were seen for clinical or other psychological outcomes. There were 13 (intervention: n=8; control: n=5) serious adverse events reported during the trial, although none were study related.

**Table 2:** Adherence to individual behaviors at baseline, 3 months, and 6 months.

Individual behavior		Intervention, n (%) (n=61)	Control, n (%) (n=62)
**Baseline**			
	Nonsmoker	49 (80)	51 (82)
	Nonharmful alcohol intake	53 (87)	53 (86)
	Physically active	17 (28)	7 (11)
	≥5 Fruit and vegetable intake	12 (20)	15 (24)
**3 months**			
	Nonsmoker	52 (85)	53 (86)
	Nonharmful alcohol intake	56 (92)	53 (88)
	Physically active	21 (34)	10 (16)
	≥5 Fruit and vegetable intake	33 (54)	18 (29)
**6 months**			
	Nonsmoker	51 (84)	55 (89)
	Nonharmful alcohol intake	53 (87)	56 (90)
	Physically active	19 (31)	15 (24)
	≥5 Fruit and vegetable intake	29 (48)	15 (24)

**Table 3:** Baseline and 6-month secondary outcomes.

Outcome			Intervention, mean (SD) (n=61)		Control, mean (SD) (n=62)		Adjusted difference (95% CI) at 6 months	*P*
			Baseline	6 months	Baseline	6 months		
**Clinical outcomes**								
	BMI		31.0 (6.4)	30.3 (5.4)	28 (4.2)	28.1 (4.4)	–0.10 (–0.56 to 0.35)	.66
	Waist-to-hip ratio		0.98 (0.07)	0.97 (0.06)	0.95 (0.07)	0.94 (0.07)	0.01 (–0.01 to 0.02)	.29
	**Blood pressure (mm Hg)**							
		Systolic	131 (17)	136 (20)	129 (26)	135 (16)	0.09 (–6.43 to 6.61)	.98
		Diastolic	78 (11)	79 (11)	75 (11)	79 (10)	–0.24 (–3.86 to 3.38)	.90
	**Cholesterol (mmol/L)**							
		Total	4.6 (1.2)	3.6 (0.7)	4.3 (1.2)	3.8 (1.1)	–0.29 (–0.61 to 0.03)	.08
		HDL	1.1 (0.3)	1.1 (0.3)	1.1 (0.3)	1.2 (0.4)	–0.04 (–0.15 to 0.07)	.51
		LDL	2.7 (1.3)	1.7 (0.6)	2.4 (1.0)	1.9 (0.8)	–0.25 (–0.49 to 0.01)	.053
	CVD risk probability			7.9 (3.4)		8.1 (3.3)	–0.27 (–1.58 to 1.04)	.68
	Medication adherence^a^			7.3 (0.9)		6.8 (1.2)	0.58 (0.19 to 0.97)	.004
**Psychological outcomes**								
	Overall illness threat		41.8 (12.3)	32.7 (11.2)	39.8 (11.6)	32.1 (12.6)	–0.4 (–4.18 to 3.35)	.83
	Hospital anxiety		6.3 (3.9)	5.8 (3.5)	5.5 (3.5)	4.4 (2.9)	1.18 (0.28 to 2.08)	.01
	Hospital depression		4.3 (3.3)	2.8 (2.8)	3.8 (2.3)	2.5 (2.2)	0.08 (–0.71 to 0.87)	.84
	Overall self-efficacy		7.6 (1.6)	8.1 (1.48)	7.9 (1.4)	8.3 (1.2)	–0.07 (–0.47 to 0.33)	.73

^a^Use of the MMAS is protected by US copyright laws. Permission for use is required. A license agreement is available from Donald E Morisky, ScD, ScM, MSPH, Professor, Department of Community Health Services, UCLA School of Public Health, 650 Charles E Young Drive South, Los Angeles, CA 90095-1772, United States.

### Intervention Fidelity and Acceptability

All but one participant randomized to the intervention group received the Text4Heart program. Intervention participants reported high fidelity to the text messaging component; 52 of 61 (85%) participants reported reading all their text messages. Nearly all participants sent in at least one step count text response (58/61, 95%) with a mean of 15 (SD 8.7) step count replies per participant over 24 weeks. A total of 23 participants sent in questions or comments to the study team via text (23/61, 38%). The vast majority of participants (55/61, 90%) felt using text messaging was a good way to deliver the Text4Heart program. Most felt that the 24-week program was the right length (48/61, 79%) and that we sent the right number of text messages (51/61, 84%). Only 5 of 61 participants (8%) felt we sent too many messages.

Less than half of participants (26/61, 43%) felt using a website was a good way to deliver the Text4Heart program. Website use data showed that 75% of participants (46/61) logged onto the website at least once during the intervention period. The number of visits to the website per person ranged from 0 to 100 (median 3) over the 6-month intervention period. Two participants reported not using the website because they did not know how to use it. Despite the lack of website use, nearly all participants (55/61, 90%) would recommend the Text4Heart program (both text message and Web) to other people who have had a heart event. Most participants felt the program helped them learn about (47/61, 77%) and recover (51/61, 84%) from their heart event. More detail on participants’ perceptions about Text4Heart can be found in Multimedia Appendix 3.

## Discussion

The Text4Heart intervention improved adherence to lifestyle behaviors at 3 months when compared to usual care alone (control), although the size of effect was not significantly retained at 6 months. A treatment effect was also observed for medication adherence. This study is one of the first to demonstrate a positive effect of an SMS-based intervention on multiple lifestyle behaviors. These findings highlight the potential utility of this approach to augment existing services in people with CHD. Strengths of the study included the RCT design, minimal loss to follow-up, high fidelity to the text messaging feature of the intervention, and the use of a composite health behavior score. A composite score allowed for a clear understanding of the intervention’s overall impact without the risk of increasing type 1 error rate [22,45].

The Text4Heart intervention was delivered in addition to usual care, which included inpatient CR and encouragement to attend phase II outpatient CR. Because both groups had similar phase II CR attendance, it appeared receiving simple text messages resulted in greater lifestyle change at 3 months. This study was powered to detect a large effect (25% difference between groups) at 6 months, yet the observed difference at this end point was 14%. A small improvement in adherence to multiple lifestyle behaviors may still have clinical relevance. To detect an effect of this magnitude (14%-15%), a post hoc sample size calculation indicated that 400 participants would be needed; thus, a larger study is warranted to examine any sustained effects of such an intervention.

Small changes to the Text4Heart program may also help boost the effects from 3 to 6 months. Relapse in unhealthy behaviors may have occurred when the intensity of our text messaging decreased. Relapse prevention and coping content should be delivered to re-engage those who drop off. Relapse prevention has been investigated in smoking cessation trials [46]; however, few studies have reported on maintenance of other healthy behaviors and strategies to prevent relapse [47] and this remains an area of future research.

### Limitations

Although treatment allocation was concealed before randomization, a limitation of this trial was that the outcome assessors were not blinded (participants were randomized at the conclusion of baseline visits by the same outcome assessors who conducted follow-up visits at 6 months). In addition, the primary outcome measure was self-reported so recall bias is possible, although validated questionnaires were used where feasible. The composite score did not capture all aspects of behavior; however, we felt it was appropriate because it is difficult to measure the multiple outcomes of CR. Objective clinical measures provided additional information associated with behavior changes; however, due to the short follow-up these findings were exploratory in nature. Another limitation was that the findings may not be transferable to other populations because our sample was predominantly New Zealand European, earned higher than the average yearly income, and were generally text message and computer literate.

### Comparisons With Other Work

Our findings extend previous research supporting the use of text message-delivered interventions to promote behavior change in people with CHD [19,48]. Text4Heart is one of the few to intervene on and measure multiple behaviors, mirroring traditional CR, which focuses on all potential behavioral risk factors. Intervening on multiple behaviors simultaneously has the potential to maximize the impact on an individual’s health and may also be more cost-effective than addressing one behavior at a time; however, it is unknown whether changing behaviors sequentially is more effective than simultaneously because few studies have compared the 2 approaches [22].

We found a positive treatment effect on medication adherence supporting other text message interventions shown to improve antiplatelet medication adherence among CHD patients [21]. The Text4Heart program incorporated several essential intervention components needed for improved medication adherence, namely, patient knowledge, counseling, and self-monitoring [49]. Future iterations might encourage greater patient counseling because the 2-way text message communication option in this study was underutilized, which may lead to stronger outcomes. The greater medication adherence score and the increased servings of fruit and vegetables observed (a component of the health behavior score) may have contributed to lower LDL cholesterol among the intervention group at 6 months. A larger trial with longer follow-up is needed to determine whether the Text4Heart intervention can make a clinically significant difference on LDL cholesterol levels.

An unexpected outcome was the lower level of anxiety observed in the control group. Both groups’ mean anxiety score was in the normal category (0-7) and decreased from baseline to 6 months. A possible explanation for the effect on anxiety might be that receiving text messages about one’s disease may lead to more anxiety. Previous research has shown that confronting a CHD diagnosis elicited negative emotions in the short term, but improved health in the long term [50]. Longer follow-up is needed to see if the difference observed in Text4Heart persisted over time.

No differences were found between groups in the self-efficacy or illness perception constructs. Both groups had high scores at baseline, which may have led to a ceiling effect. Future studies should focus on patients who have low self-efficacy to change behavior.

### Implications for Clinical Practice and/or Research

The Text4Heart intervention was a simple package of text messaging and a website; however, because the website was used infrequently and usual care CR attendance was similar across groups, receiving text messages was likely the predominant contributor to the observed effects. A text messaging program could be easily incorporated into existing CR, either as an alternative option for those unable to attend center-based programs or as an add-on to extend current services. It may be that earlier program commencement after diagnosis and the longer duration of Text4Heart helped facilitate and maintain behavior change. There is potential for this type of program to reach underserved populations and those with access-to-care barriers, such as patients living in deprived areas or developing nations. Future research should be undertaken with more diverse samples to determine if such an intervention can reduce health inequalities.

Text4Heart was relatively simple to develop and use. A similar study delivering exercise-based CR via text message was considered to be cost-effective for walking and leisure-time physical activity [19]. Apps [48] and the use of biofeedback and wearable sensors have also been used to deliver CR. Apps and sensor technologies may result in stronger effects because they can allow for greater individual tailoring and improved 2-way communication with health care providers; however, such technology are associated with greater financial and time costs. More research is needed to compare outcomes of text messaging and “appified” approaches before investing significant resources into complex interventions.

### Conclusion

Receiving a simple text message-delivered CR intervention in addition to usual care had a positive effect on adherence to multiple lifestyle behavior changes in New Zealand adults with CHD at 3 months; however, the effect had attenuated by 6 months. A larger study with longer follow-up is needed to determine whether these behavior changes can result in clinically significant outcomes.

## Appendix

Multimedia Appendix 1 eHealth CONSORT checklist form.

Multimedia Appendix 2 Participant information sheet and informed consent form.

Multimedia Appendix 3 Participant perceptions of the Text4Heart intervention.

## Abbreviations

CHD: coronary heart disease
CR: cardiac rehabilitation
LDL: low-density lipoprotein
RCT: randomized controlled trials
SMS: short message service
CVD: cardiovascular disease
