# Supplementary material for: Text Message and Internet Support for Coronary Heart Disease Self-Management: Results From the Text4Heart Randomized Controlled Trial
Source: J Med Internet Res. 2015 Oct 21;17(10):e237. doi: 10.2196/jmir.4944 (PMC4642389; doi:10.2196/jmir.4944)
Supplement: Multimedia Appendix 2 [file jmir_v17i10e237_app2.pdf]

Investigator: Ms Leila Pfaeffli, University of Auckland
Telephone: (09) 923-4728

CONSENT TO TAKE PART IN A RESEARCH STUDY: TEXT4HEART

We would like to invite you to take part in the **Text4Heart Study**. If you have any questions please feel free to ask the researcher at any time. **Please read the booklet called "Participant Information for Clinical Studies."**

<u>Purpose of the study</u>

We are inviting you to join this study because you have a diagnosis of acute coronary syndrome (myocardial infarction [MI] or unstable angina) or percutaneous coronary intervention procedure. The study is looking at whether delivering a cardiac rehabilitation programme via mobile phone and the internet alongside usual care rehabilitation is more effective than taking part in cardiac rehabilitation alone.

<u>Where will the study be held?</u>

Participants for this study will be identified by research nurses at Auckland City and Waitemata District Board Hospitals. The face-to-face visits will take place at hospitals or at a convenient location.

<u>About the study</u>

We plan to recruit 120 people over an 8-month period. You will be in the study for 24 weeks, and will attend two visits: an initial visit and another 24 weeks later. The entire study will run for 15 months, from May 2013 to August 2014. If you agree to take part in the study, you will be randomly allocated (like the toss of a coin) to one of two groups:

- **Intervention group:** Everyone in this group will receive a personalised cardiac rehabilitation programme delivered by text message and the internet over 24 weeks. The programme will inform participants about medication, healthy eating, reducing stress, exercising, and stopping smoking. Participants will be encouraged to attend standard cardiac rehabilitation programmes.

- **Control group:** Participants will be encouraged to attend standard cardiac rehabilitation programmes.

Who can take part in this study?

You can take part in this study if you:

- Have a diagnosis of coronary heart disease (myocardial infarction [MI], angina, or unstable angina) or percutaneous coronary intervention procedure
- Have access to the internet
- Can understand and read English
- Are aged 18 years or over
- Are able to provide written consent to take part

You cannot take part in this study if you have:

- Significant exercise limitations
- Life threatening co-existing disease
- Untreated ventricular tachycardia or severe heart failure

Study design

Please look at the picture below which will give you an idea about the study visits.

What will I be asked to do?

- If you agree to take part in the study you will be asked to sign a consent form at the first face-to-face meeting with the researcher.
- At each face-to-face meeting we will ask you some questions about your lifestyle. We will measure your blood pressure, height, weight and waist circumference. You do not have to answer all the questions, and you may stop the

survey at any time. Each meeting will take about 45 minutes. At your final visit we will also measure your blood cholesterol.

- At the end of the study, you will be given a $20 food or petrol voucher as reimbursement for your time in the study.

Blood samples

At your follow-up visit, you will be asked to complete a fasting point of care blood test, to measure your cholesterol. Fasting requires you to not eat anything for 10-16 hours, and is best done overnight. We will do the face-to-face visits first thing in the morning. During your fasting period you may drink water and take any medications you would normally take.

A point of care blood test involves taking blood from your finger. The research staff is extensively trained in this skill. All of the equipment is sterile. If you feel uncomfortable, please do not hesitate to inform the researcher. You may prefer to lie down if you are feeling faint or nervous.

The researcher will cleanse the skin with an alcohol swab and then place a lancet next to your finger. You will feel a sharp pinch. The lancet is removed and a few drops of blood will be collected into a capillary tube (34 microlitre=less than a mililitre). The sample is then inserted into a portable device that measures your cholesterol. You will receive the results immediately. The blood sample is then disposed of into a biohazard waste container, or you may keep the sample if you wish.

After the blood has been drawn pressure should be applied to the site for at least three minutes to minimise bleeding. Once bleeding has stopped, a bandaid will be applied. If you are aware of any allergic reactions to alcohol swabs or tapes please let the researcher know and we will find an alternative.

What if I don't want to participate?

Your participation in this study is entirely voluntary (your choice). You do not have to take part.

Potential benefits

Cardiac rehabilitation is an essential part of the care of people with heart disease. Indeed, cardiac rehabilitation has been shown to slow or reverse the progression of cardiovascular disease in people who have had a heart event. There are many potential benefits to being able to deliver a cardiac rehabilitation programme by mobile phone and the internet. Programmes are flexible and can be tailored to specific cultural, age group and health needs. Messages can be delivered at anytime, anywhere, regularly and frequently. Your responses will help researchers to see whether providing a cardiac rehabilitation programme using mobile phones and the internet may benefit people with cardiovascular disease in the future. If the programme is useful, researchers will look at the possibility of delivering other outpatient programmes by mobile phone and the internet. Participants may benefit from extra monitoring they receive as part of this study.

Potential risks and discomforts

Cardiac rehabilitation is standard care for people recovering from cardiovascular disease. There are no anticipated risks from taking part in this programme over and above the risks associated with taking part in routine cardiac rehabilitation which

may include engaging in everyday physical activity, modifying diet, and stopping smoking.

As a result of overnight fasting, you may experience some discomfort associated with being hungry. As with all blood tests, there is a very small risk of infection. However, as stated above, this risk is minimised by having appropriately certified experimenters taking the samples, and following approved hygiene protocols.

If you were injured in this study, which is unlikely, you would be eligible for compensation from ACC just as you would be if you were injured in an accident at work or at home. If you have private health or life insurance, you may wish to check with your insurer that taking part in this study won't affect your cover.

Your rights and responsibilities are described in the booklet "Participant Information for Clinical Studies". Please read this and make sure all your questions are answered before you sign this consent form.

The study has been approved by the Health and Disability Ethics Committee [13/NTA/6], Auckland DHB Research Review Committee [A+5816], and the Waitemata Awhina Research and Knowledge Center [RM 0980712441]

What to expect at each visit:

	Randomised (face-to-face)	Intervention group only: Daily text for weeks 1-24 and 3 month call	24 weeks (face-to-face)
**How long will it take?**	45 minutes	~30 sec per text, ~5 min for the phone call	45 minutes
**Study explained, consent obtained**	✓		
**Inclusion and exclusion criteria checked**	✓		
**Blood sample collected**			✓
**Physical measurements**	✓		✓
**Questions about your lifestyle behaviours**	✓		✓

Thank you for reading this information leaflet

CONSENT

I have read and I understand this information sheet and the booklet on my rights. I have had the opportunity to discuss this study. I am satisfied with the answers I have been given and that I have had sufficient time to consider whether to participate in the study. **YES/NO**

I consent to my blood sample being taken **YES/NO**

I would like my blood sample returned to me after analysis **YES/NO**

I wish to receive a copy of the results. **YES/NO**

I consent to take part in this study

**Participant Name (Print)**		**Participant Signature**	
**Date**			

I have given a verbal explanation of the research project to the participant, and believe that the participant understands the study and has given informed consent to participate.

**Researcher's Name (Print)**		**Researcher's Signature**	
**Date**			
