# Supplementary material for: Text Message and Internet Support for Coronary Heart Disease Self-Management: Results From the Text4Heart Randomized Controlled Trial
Source: J Med Internet Res. 2015 Oct 21;17(10):e237. doi: 10.2196/jmir.4944 (PMC4642389; doi:10.2196/jmir.4944)
Supplement: Multimedia Appendix 3 [file jmir_v17i10e237_app3.pdf]

Appendix 2. Intervention participants' perceptions on Text4Heart program

Nearly all participants felt the Text4Heart program helped them change their behaviour (47/61; 77%), in particular becoming more physically active (39/61; 64%), eating more fruit and vegetables (37/61; 61%); eating less saturated fat (34/61; 56%) and salt (26/61; 43%), and taking medications more regularly (20/61; 33%). Table 1 displays the content analysis of open-ended questions regarding participant perceptions of the intervention.

Table 1. Content analysis of open-ended survey responses (n=56)

I liked the program because it:	Number of comments
Provided social support	11
Made me accountable	3
Was motivating	8
Was encouraging	6
Was educational	5
Was a good reminder	21
Suited my lifestyle (texts)	4
Other	7
I disliked the program because:	
The messages were too generic	3
The messages were irrelevant	3
The messages were too repetitive	2
I'm not interested in blogging	2
Technical issue	4
Other	3

The program can be improved by:	
Personalizing the messages	4
Adding personal contact	2
Shortening the length of the program	2
Adding a follow-up program of SMS	14
Other	9
